# The Influence of Social Support on Physical Activity in Chinese Adolescents: The Mediating Role of Exercise Self-Efficacy

**DOI:** 10.3390/children7030023

**Published:** 2020-03-20

**Authors:** Zhanbing Ren, Linlin Hu, Jane Jie Yu, Qian Yu, Sitong Chen, Yudan Ma, Jingyuan Lin, Lin Yang, Xiaoyun Li, Liye Zou

**Affiliations:** 1 Department of physical education, Shenzhen University, Shenzhen 518061, China; rzb@szu.edu.cn (Z.R.); hulinlin528@163.com (L.H.); sitongchen@szu.edu.cn (S.C.); 2 Department of Sports Science and Physical Education, The Chinese University of Hong Kong, Shatin, New Territories, Hong Kong, 999077, China; jieyu0203@gmail.com; 3 Exercise and Mental Health Laboratory, School of Psychology, Shenzhen University, Shenzhen 518061, China; yuqianmiss@163.com (Q.Y.); linjingyuan921@126.com (J.L.); 4 Jilin Institute of Sport Science, Changchun 130022, China; yudanma@sina.cn; 5 Department of Cancer Epidemiology and Prevention Research, Alberta Health Services, Calgary, AB T2S 3C3, Canada; Lin.Yang@albertahealthservices.ca; 6 Departments of Oncology and Community Health Sciences, Cumming School of Medicine, University of Calgary, Calgary, AB T2N 4Z6, Canada; 7 Zhongshan No.2 High School, Shenzhen 518061, China; lixiaoyun601@163.com

**Keywords:** social support, adolescents, physical activity, exercise self-efficacy, mediating role

## Abstract

The purpose of this study was to examine the associations of social support and self-efficacy with physical Activity (PA) and the mediating effect of self-efficacy on the relationship between social support and PA in Chinese adolescents. Participants included a total of 2341 Chinese adolescents (aged 12.75 ± 1.46 years). Self-reported instruments, including the physical activity questionnaire for adolescents, the social support revalued scale and the exercise self-efficacy scale, were used to measure physical activity, social support and exercise self-efficacy. Results showed that social support (r = 0.29, *p* < 0.05) and exercise self-efficacy (r = 0.43, *p* < 0.05) were significant and positive predictors of PA among Chinese adolescents, and exercise self-efficacy was a significant mediator in the relationship between social support and PA (standardized effect size = 0.15, *p* < 0.001). Such findings were evident with similar patterns in both male and female adolescents. The findings of this study have indicated the importance of social support and exercise self-efficacy on PA promotion in adolescents, which will aid the development of effective interventions in this population.

## 1. Introduction

Physical activity (PA) refers to any movement of skeletal muscles that requires energy expenditure. Regular participation in PA promotes physical health (e.g., bone growth and development, muscular strength, cardiovascular fitness) [1] and reduces the risk of noncommunicable diseases [2,3]. Moreover, it benefits psychological and cognitive development, optimizes social cognition, enhances social ability, strengthens self-confidence and facilitates social interaction in adolescents [3,4]. Developing a habit for regular PA in childhood and adolescence is important for both physical and mental health. However, globally almost 80% of adolescents do not meet the recommended amount of PA [5]. Chinese adolescents were also found to be physically inactive, with only 10.3% performing moderate–to–vigorous PA for more than 60 min per day on five days a week, as recommended [6]. Furthermore, it has been shown that PA participation of Chinese adolescents varies by gender, with males more likely to be physically active than females [7,8,9]; in this case, gender is usually considered in the research to investigate correlates of PA.

PA is a complex behavior influenced by various factors, from personal, societal to environmental levels [10]. Many previous studies have investigated PA correlates from a personal level, such as self-efficacy; however, less research has been carried out on the correlations between PA and social factors, including the influence from parents and peers [11]. Social cognitive theory suggests that social support could be a decisive factor for adolescent PA [12], and studies have indicated that a chain of decision-making behaviors in adolescents to increase their involvement in PA, namely to start, to continue, to stop and to quit, are influenced by social support [13]. Social support refers to the respect, caring and help given by an important individual or another group that is close to an individual. These include parental involvement [14], parental support [15], peer support [16] and family cohesion [17]; among these, support from parents and peers are crucial for adolescent PA [18,19] as adolescents that have received greater support from their parents and friends, are more likely to participate in PA [20]. Positive parental support can encourage adolescents to participate in more PA [21], help establish correct attitudes and values towards PA, and motivate them to become directly involved in PA [22]. Notably, as children enter into adolescence, peer support becomes more important than family support. It is believed that the influence of peers increases along with age. Peer support is defined as an individual who has shared similar interests or planned behaviors with others in daily life [23]. The presence of peers is not simply associated with an increased amount of PA participation [24], but can also influence PA indirectly through self-efficacy in teenagers [25]. The formation and maintenance of a friendship network that promotes parental support and PA may play an important role in controlling the decline in adolescent PA [26]. In considering previous studies on adolescent PA and social support, it appears that social support has an impact on adolescent PA. However, scholars have not yet reached an agreement on whether the impact is direct or indirect. 

Self-efficacy is identified as a key component of social cognitive theory as it can influence healthy behaviors, including PA, directly or indirectly [27]. In this article, self-efficacy is defined as people’s confidence in self-organizing and the ability to perform PA in the face of various difficulties. Research has suggested that self-efficacy is one of the most reliable factors for PA [28], and there is a strong relationship between adolescent PA and exercise self-efficacy [29]. It has been found that self-efficacy plays a positive role, especially for adolescent girls [30]. Self-efficacy can indirectly encourage adolescents to perform PA more actively by promoting the advancement of PA behavioral changes [31,32]. Moreover, previous research indicated the interrelation between self-efficacy and social support in adolescents. For example, it has been found that parental support can predict exercise self-efficacy, and exercise self-efficacy also exerts influence on adolescent health and well-being behaviors [33]. 

Taken together, the importance of social support and self-efficacy for PA is confirmed [34]. However, it remains elusive about the associations of social support and self-efficacy with PA and how self-efficacy mediates the association between social support and PA participation in adolescents, especially among the Chinese population. Thus, the purpose of this study was to examine the associations of social support and self-efficacy with PA in Chinese adolescents, and the mediating effect of self-efficacy on the relationship between social support and PA. As per the tenets of previous research [35,36], we hypothesized that: (1) there would be significant associations among PA, social support and exercise self-efficacy in male and female adolescents; (2) exercise self-efficacy would be a significant mediator in the relationship between social support and PA in adolescents; that is, social support would have a direct impact on PA and an indirect impact on PA, through exercise self-efficacy.

## 2. Materials and Methods

### 2.1. Participants

In this study, a total of 2500 adolescents aged between 12 and 17 years old were initially recruited from 10 mainstream schools (including 6 junior high schools and 4 high schools) in Shenzhen, China. Data from 159 participants were considered invalid due to omitted responses in the survey. Thus, data from 2341 (93.6%) of the 2500 participants were eventually included for data analysis, which included 1178 males and 1163 females. This study was approved by the research ethics committee of Shenzhen University. All participants took part in this study voluntarily, with written informed consent from their parents or guardians.

### 2.2. Procedure

Data collection was conducted at participating schools during school time, in September 2019, by trained research assistants. Knowledge about key concepts (e.g., PA, self-efficacy) related to the outcomes was explained to the participants before they filled in the survey. All questions raised from the participants were answered and solved to avoid any misunderstandings. The survey contained three questionnaires and took approximately 15 min for each participant to complete. 

### 2.3. Instruments

The physical activity questionnaire for adolescents (PAQ-A) was used to evaluate the PA level. It was specifically developed by Canadian scholars for adolescents, to measure their PA over the previous 7 days [37]. The original version has been adapted and validated in Chinese [38], with 0.85 and 0.82 in the internal consistency reliability and the test-retest reliability, respectively. Similarly, the PAQ-A of the Chinese version has 9 questions (Q) with a 5-point Likert Scale. Q9 is typically used as a screening question to determine health status (a bad cold, period, broken bones or any other illness) of selected participants that affect their PA participation. If someone reported any condition, his or her data would not be used for data analysis. The mean score of the remaining eight questions was calculated and included for data analysis. In this study, results from confirmatory factor analysis included Chi-squared(X^2^) = 81.128, df = 18, X^2^/df = 4.507, Root Mean Square of Approximation (RMSEA) = 0.039, Goodness of Fit Index (GFI) = 0.992, Adjusted Goodness of Fit Index (AGFI) = 0.983, Normed Fit Index (NFI) = 0.990 and Comparative Fit Index (CFI) = 0.992. The Cronbach α value was 0.87 for all questions in the PAQ-A. The item-total correlation was 0.207–0.917 (*p* < 0.01).

The exercise self-efficacy scale (ESES) was used to assess exercise self-efficacy. The original version [39] by Marcus, Selby and Niaura (1992) was revised and validated with high internal consistency (α = 0.82) [40], which was supported by the results of the present study (the internal consistency reliability of Cronbach α = 0.91). The Chinese version of ESES consists of 18 questions categorized into four domains (i.e., physical factor, activity factor, mental factor and conflict factor) [40]. Each question involves a 5-point Likert scale reflecting different degrees of confidence to regularly exercise, with 1 = “not confident” and 5 = “very confident.” The mean score of all items was calculated as the outcome measure. In the present study, results from confirmatory factor analysis included X^2^ = 1427.17, df = 116, X^2^/df = 12.30, RMSEA = 0.07, GFI = 0.93, AGFI = 0.90, NFI = 0.96 and CFI = 0.97. The Cronbach α value was 0.87 for all items of ESES and was 0.85, 0.91, 0.91 and 0.90 for the domains of physical factor, activity factor, mental factor, and conflict factor, respectively. The item-total correlation is 0.668–0.838 (*p* < 0.01). 

Social support was measured using the social support revalued scale (SSRQ) [41]. The SSRQ consists of 10 questions within 3 domains (subjective support, objective support and the utilization of support). Objective support refers to the physical and actual support received by participants, including financial or material assistance, social networks and parental involvement; subjective support refers to emotional support experienced by participants, including being respected by the society, being supported and understood, and degree of satisfaction, which is closely related to the subjective feeling of individuals; utilization of support refers to individual’s active usage of various social support, including through sharing feelings, asking for help and participating in activities. Because the participants in this study were adolescents, the description of some items was adjusted accordingly. For example, “the relationship with neighbors” was replaced with “the relationship with classmates or roommates”. For Q1–Q4 and Q8–Q10, participants were required to choose only one answer for each question, with Option 1, 2, 3 and 4 being scored 1, 2, 3 and 4 points, respectively. For Q5, the scores for 4 options were added up. “No support” scored 1 point and “full support” 4 points. For Q6 and Q7, participants earned 0 points if they selected “no source”; if “the following source” was selected, they scored the number of sources selected. The total score was calculated by adding up the scores of 10 questions as the outcome measure. Higher scores indicated more social support. In this study, results from confirmatory factor analysis included X^2^ = 180.59, df = 29, x2/df = 6.23, RMSEA = 0.05, GFI = 0.99, AGFI = 0.97, NFI = 0.97 and CFI = 0.98. The Cronbach α value was 0.66 for all items of SSRQ and was 0.85, 0.91, 0.91 and 0.90 for the domains of subjective support, objective support and the utilization of support, respectively. The item total correlation was 0.21–0.64 (*p* < 0.01).

### 2.4. Data Collection and Analysis 

Using the software of SPSS 24.0, descriptive statistics; e.g., the mean, standard deviation (SD), and 95% confidence interval (CI) of all outcomes were explored. Gender differences in all outcomes were examined using the independent t-tests. The correlations between PA, exercise self-efficacy and social support were examined using the Pearson’s partial correlation coefficients after controlling for age. The prediction effects of exercise self-efficacy and social support on PA level were examined using linear regression analyses after controlling for age and gender. Using the software of AMOS 22.0, the bootstrap method was used to test whether exercise self-efficacy was a mediator in the relationship between social support and PA level among 2000 participants who were randomly selected from the total sample. The significance level was set as *p* < 0.05. 

## 3. Results

### 3.1. Examination of Common Method Bias 

Common method bias occurs when variations in responses are attributed to measurement method instead of the constructs the measures are assumed to represent. In this study, social support, PA level and exercise self-efficacy were all collected by self-report, which may lead to common method biases (CMB). To identify whether CMB existed, Harman’s one-factor test, as a useful technique, was used in this study [42]. All the questions of PA, social support and exercise self-efficacy were analyzed using unrotated factor analysis in SPSS. The variance explained the rate of the first common factor was 36.60% (<40%). Therefore, there was no severe CMB. 

### 3.2. PA, Exercise Self-Efficacy and Social Support in Male and Female Adolescents

Descriptive statistics of PA, exercise self-efficacy and social support are presented in Table 1. There were significant gender differences in the PA level score (t = 9.97, *p* < 0.001) and exercise self-efficacy (t = 3.35, *p* < 0.01), and males scored higher than females in both outcomes (Table 1). The results indicated that males had a higher PA level and better exercise self-efficacy than females. However, no significant gender difference was found for social support (*p* > 0.05) (Table 1).

### 3.3. Associations Among PA, Exercise Self-Efficacy and Social Support 

As presented in Table 2, there were significant and positive correlations between each outcome (i.e., PA level, exercise self-efficacy and social support) after controlling for age. A similar pattern of the associations among PA level, exercise self-efficacy and social support was found in both females and males (Table 2). A regression analysis was performed with social support and exercise self-efficacy, as independent variables, and PA as a dependent variable, after controlling for age and gender. The results showed that exercise self-efficacy and social support were significant predictors for the PA level (both *p* < 0.001), accounting for 23.20% and 26.70% of the variance, respectively (see Table 3).

### 3.4. The Mediating Role of Exercise Self-Efficacy

Structural equation modeling was applied to test whether exercise self-efficacy mediated the association between PA level and social support. The model consisted of three latent variables: PA level, social support and exercise self-efficacy. When the data relating to social support and PA level were initially put in the model, results showed that common fit indices were good with X^2^/df = 3.40, TLI = 0.97, CFI = 0.97 and RMSEA = 0.04. This was followed by adding exercise self-efficacy as a mediator in the model, which resulted in acceptable fit indices with X^2^/df = 4.87, TLI = 0.95, CFI = 0.96 and RMSEA = 0.04.

**Figure 1:**
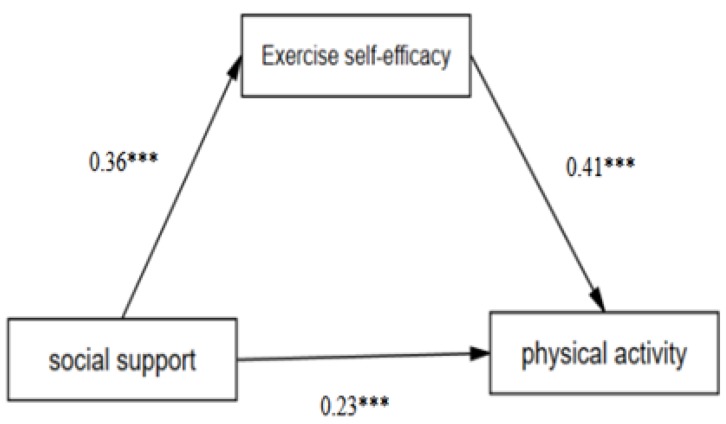
The mediating effect of exercise self-efficacy. (Notes: *** means *p* > 0.001.)

**Figure 2:**
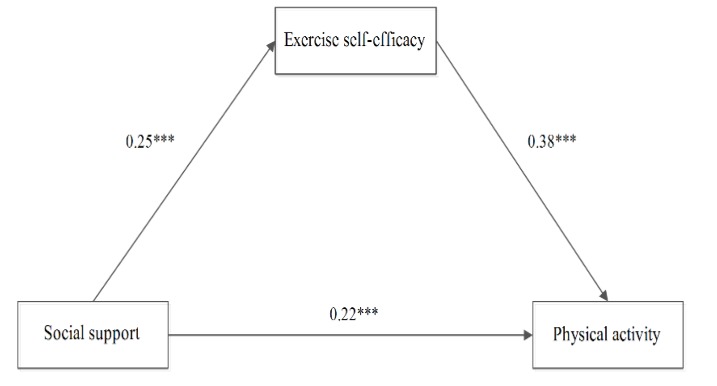
The mediating effect of exercise self-efficacy in boys. (Notes: *** means *p* > 0.001.)

**Figure 3:**
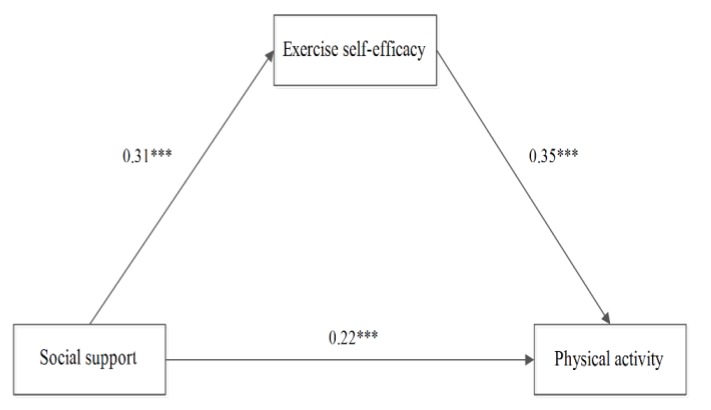
The mediating effect of exercise self-efficacy in girls. (Notes: *** means *p* > 0.001.)

The results showed a significant mediation effect of exercise self-efficacy on the association between PA and social support among all participants (standardized effect size = 0.15, 95% CI 0.13 to 0.18, *p* < 0.001), as shown in Figure 1. Notably, its mediating effect accounts for 38.46% of the effect of social support on PA. Similarly, a significant mediation effect of exercise self-efficacy on the association between PA and social support was also observed in both males (standardized effect size = 0.09, CI 95% 0.07 to 0.13, *p* < 0.001), as shown in Figure 2, and females (standardized effect size = 0.11, CI 95% 0.08 to 0.14, *p* < 0.001), as shown in Figure 3. 

## 4. Discussion

### 4.1. PA, Social Support and Exercise Self-Efficacy 

The results of this study showed a significant gender difference in adolescent PA. Males engaged in a higher level of PA than females; this may be owing to the traits and characteristics of males. The PA gap between males and females emerges at an early stage. As age increases, females have reported low levels of enjoyment in relation to exercise, and consequently, confidence in athletic ability is likely reduced. In a study on adolescent PA, 61.5% of males reached the recommended PA level, while the figure for females was 36% [43]. Another study also pointed out that the PA frequency and intensity of adolescent females were lower than those of males [44]. Moreover, the present study found that there was no significant gender difference in social support for adolescents. This is consistent with the study by Chen et al. [45]. In addition, the present study found a significant gender difference in exercise self-efficacy. Compared with female adolescents, males usually have higher self-evaluation and judgment on whether they have successfully completed exercise, achieved good exercising performance and met the goals. Therefore, effective interventions on improving the PA level and exercise self-efficacy of adolescents should be developed, especially in girls. 

### 4.2. The Influence of Social Support and Exercise Self-Efficacy on Adolescent PA

In this study, social support played a positive role in adolescent PA, which was consistent with previous studies [46,47]. It has been shown that general social support from parents and peers was positively correlated to the overall PA level. In general, it is believed that adolescents who received more social support participate in a higher level of PA especially when the support is provided by parents, siblings and friends. Parents can encourage their children to participate in PA in various ways, such as providing equipment and transportation [47]. Also, friends tend to provide social support by inviting and participating in PA [48]. Friends are a source of social support, indicating that the association between PA and social support is the most consistent. 

The results of this study indicated that exercise self-efficacy has a significant positive predictive effect on adolescent PA, which is consistent with previous study findings [49,50]. Adolescents with better exercise self-efficacy participate in a higher level of PA. One previous study on the PA and exercise self-efficacy in 483 Spanish adolescents found a strong correlation between adolescent PA and self-efficacy [51]; in addition, Wing et al. reported that self-efficacy was positively correlated with PA participation, in and out of school time, among 595 adolescents [52]. In the face of difficulties in sports, adolescents with high exercise self-efficacy may effectively regulate their own emotions, change their attitudes and be willing to make more efforts in order to overcome the difficulties, thus, the PA level is increased correspondingly. When facing stress and difficulties in sports, adolescents with low self-efficacy may easily exaggerate the difficulty and generate negative emotions such as anxiety, and thus the PA level is low. There is a significant positive correlation between the sense of exercise self-efficacy and PA, indicating that the improvement in self-efficacy leads to improved PA. Therefore, how to improve exercise self-efficacy in adolescents has become a key issue in the PA promotion in this population. 

### 4.3. The Mediating Role of Exercise Self-Efficacy

In this study, exercise self-efficacy was considered when studying the correlation between PA and social support in adolescents. We found that exercise self-efficacy was a significant mediator of the relationship between social support and adolescent PA. This finding was consistent with previous findings; for example, Verloigne et al. [53] conducted a study to explore the mediating effects of self-efficacy on the association between peer/parental support and PA among 226 girls. They found that the sense of self-efficacy mediated the associations between peer/parental support and PA. The present study also found a significant positive correlation between social support and exercise self-efficacy in adolescents. Notably, social support has both a direct and indirect impact on the PA level of adolescents. 

Adolescents spend most of the exercising time in a day with their classmates. Support and recognition of their peers give adolescents a sense of belonging and can improve their self-evaluation and PA level. Adolescents are in their puberty, with “contradiction” being the main feature. In their relationship with parents, it is shown as, on the one hand, trying to escape the restrictions set by their parents and a longing for independence and freedom; and on the other hand, when they encounter difficulties, they hope to gain support from their parents. In this study, the self-efficacy of adolescents’ exercise was a powerful intermediary for social support and PA. Future investigations should pay more attention to research on the improvement of adolescents’ exercise self-efficacy. Adolescents should be provided with more PA opportunities to improve their interest in sports. The actual exercise capabilities of adolescents should also be fostered, to further increase their exercise self-efficacy.

### Limitations and Strengths of This Study 

The strengths of this study include a large sample size, random selection and standard procedures of data collection and analysis. Admittedly, this study had several limitations. Firstly, data in this study was collected from one of the most economically powerful cities in China. The sample in this study may not be representative of the general population of adolescents in China. Secondly, all measures were self-reported, which may involve recall errors and subjective bias. Thirdly, factors of socioeconomic status (e.g., family monthly income) were not collected in this study, which is worth investigating in future research. Lastly, this study was a cross-sectional study in nature and thus can not indicate cause–and–effect relationships. The associations of social support and exercise self-efficacy with PA in adolescents should be further verified in future intervention studies. 

## 5. Conclusions 

We conclude that exercise self-efficacy and social support are significant and positive predictors to PA in Chinese adolescents, and exercise self-efficacy is a significant mediator in the relationship between social support and PA. Social support can impact adolescent PA directly and indirectly through the mediating effect of exercise self-efficacy. The pattern of the associations between exercise self-efficacy, social support and PA is identical in males and females. Future PA interventions for adolescents are encouraged to incorporate strategies for improving social support and exercise self-efficacy to increase their effectiveness. 

## Figures and Tables

**Table 1:** Descriptive statistics and sex differences in PA level, exercise self-efficacy and social support. (M ± SD).

Outcome	Overall (N = 2341)	Males (*n* = 1178)	Females (*n* = 1163)	*t* Value
PA level	2.27 ± 0.75	2.42 ± 0.77	2.12 ± 0.70	9.97 ***
Exercise self-efficacy	2.92 ± 0.81	2.97 ± 0.84	2.86 ± 0.77	3.35 **
Social support	36.86 ± 6.85	36.17 ± 6.97	36.12 ± 6.74	0.17

Notes: M = mean; SD = standard deviation; PA = physical activity; ** *p* < 0.01; *** *p* < 0.001.

**Table 2:** Associations among PA level, social support and exercise self-efficacy.

	PA Level	Exercise Self-Efficacy	Social Support
**Overall**			
PA level	1		
Exercise self-efficacy	0.43 ***	1	
Social support	0.29 ***	0.27 ***	1
**Males**			
PA level	1		
Exercise self-efficacy	0.44 ***	1	
Social support	0.31 ***	0.26 ***	1
**Females**			
PA level	1		
Exercise self-efficacy	0.40 ***	1	
Social support	0.30 ***	0.29 ***	1

Notes: PA = physical activity; adjusted for age; *** *p* < 0.001.

**Table 3:** Regression analysis on the predictors of the PA level.

Variable	B	SE	β	T	F	R^2^
Exercise self-efficacy	0.33	0.02	0.36	19.37 ***	509.99	0.23
Social support	0.02	0.002	0.20	10.66 ***	113.64	0.27

Notes: *** means F is at the significance level of 0.001. Adjusted for age and gender.
